# Temporomandibular joint disorder in systemic sclerosis: a case report

**DOI:** 10.11604/pamj.2016.25.164.10432

**Published:** 2016-11-16

**Authors:** Raja Chebbi, Hanen Ben Khalifa, Monia Dhidah

**Affiliations:** 1 Unit of Orofacial Pain and Temporomandibular Dysfunction, University Dental Clinic, Monastir, Tunisia

**Keywords:** Systemic sclerosis, temporomandibular joint, pain, condylar resorption

## Abstract

Systemic sclerosis have several effects on the orofacial region such as widening of the periodontal ligament space, xerostomia and bone resorption of the mandible. We report a case of systemic sclerosis with temporomandibular joint involvement in a 45-year-old female patient accompanied by severe limited mouth opening and pain in the right and left preauricular regions and tenderness in masseter muscles with a morning stiffness of jaws.Magnetic resonance imaging showed a resorption of mandibular condylar process, with disk and joint abnormalities.

## Introduction

Systemic sclerosis (SSc) is an autoimmune disease characterised by vascular alterations and extensive fibrosis. Patients with SSc may present a Raynaud phenomenon, pulmonary hypertension, skin thickening, esophagealdysmotility, arthralgia, and renal insufficiency [1]. SSc causes several oral and maxillofacial manifestations such as masklike appearance, limited mouth opening, skin and muscular atrophy, peribuccalrhagades, thin atrophied lips, microstomia, xerostomia, widening of the periodontal ligament space, trigeminal neuralgia, telangiectasia and bone resorption of the mandible [2–5]. Few studies in the literature are published concerning the temporomandibular joint (TMJ) and masticatory muscles involvement in SSc. Most descriptions are based on individual cases or small numbers of patients with inadequate controls and do not define the relationship of oral manifestations to other manifestations of the disease [4, 6–9]. Thus, the frequency of TMJ and muscle disorders involvement in patients with SSc was poorly assessed. This case report describes the temporo mandibuar joint disorders in 45-year-old female patient with SSc, based on clinical and radiological data.

## Patient and observation

A 45-year-old women with SSc was referred to the unit of orofacial pain and temporomandibular dysfunction of the university medical dental clinic of Monastir, Tunisia, with a progressive limited mouth opening and facial pain since 2 years. A history of pain in the right and left pre-auricular regions and masticatory myalgia more pronounced in masseter muscles with a morning stiffness of jaws was depicted. These symptoms are aggravated by function. A sounds in the right TMJ were also reported. She had no history of trauma or infection of the TMJ. The patient had a medical history of limited cutanous systemic sclerosis diagnosed by an internal medicine specialist since 3 years and treated by methotrexate, colchicine, amlodipine, methylprednisolone, cholecalciferol, sulodexide, acidumfolicum and Lysinacetylsalicylas. The TMJ and muscle symptoms was noted since 1 year after her diagnosis. These symptoms have slowly worsened prompting the patient to run our specializing consultation.

**Figure 1:**
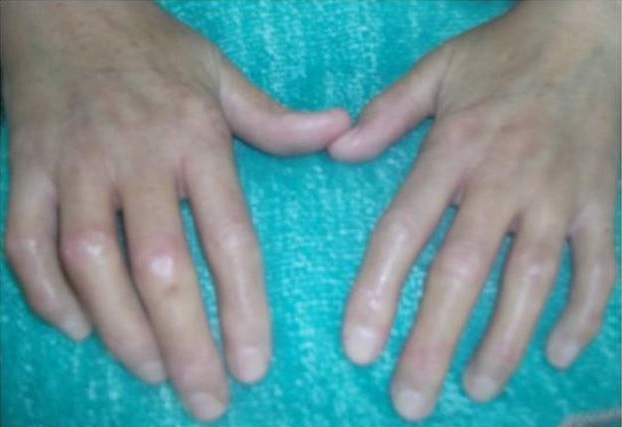
Clinical signs of scleroderma on the hands showing the flexion contracture associated with sclerodactyly and digital ulcers

**Figure 2:**
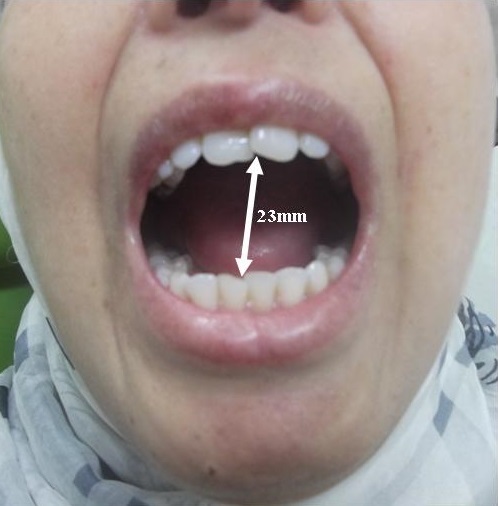
Restricted mouth opening and trajectory deviation to the right side

Physical examination of the patient revealed that SSc affected mainly her hands with a fingers movementlimitation, associated with sclerodactyly complicated by digital ulcers, moderate lung fibrosis with a flexion contracture and a claw-like deformity (Figure 1). The clinical examination of the orofacial region revealed a masklike facies with a smooth and tight facial skin with a loss of normal animation lines and a bilateral stiffness in masticatory muscles at palpation and movements. The mouth opening was limited to 23 mm and the mandible deviated to the right side (Figure 2). The other mandibular movements such as propulsion and deduction are also restricted. The palpation of TMJ region causes severe pain more pronounced in the right side and a restricted condylar movement. No joint sounds and no malocclusion are depicted

**Figure 3:**
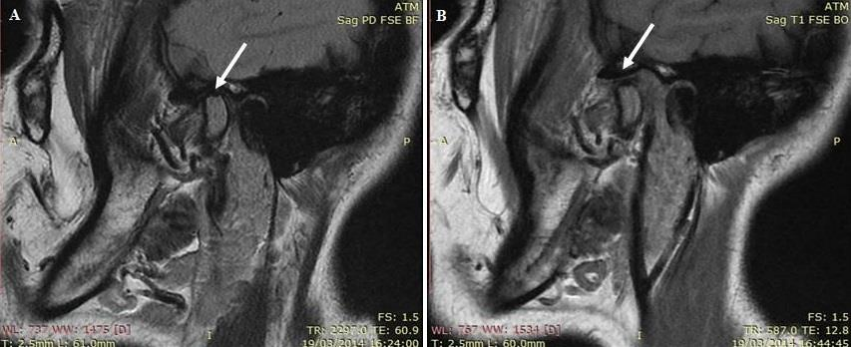
Magnetic resonance imaging (MRI) sagittal slices: A) MRI in closed-mouth position showing mandibular condylar process resorption, joint space decreased anddegenerative alterations of the disk; B) MRI in open-mouth position showing hypomobility of mandibular condylar

**Figure 4:**
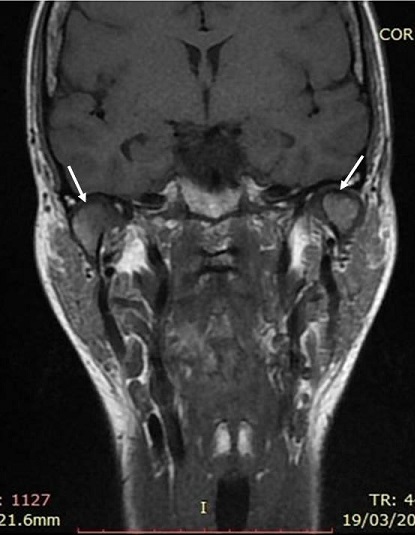
Magnetic resonance imaging (MRI) coronal slices showing bilateral subchondral geodes and thinned cortical of mandibular condylar process

Magnetic resonance imaging (MRI), in open- and closed-mouth positions, was performed to evaluate the TMJ and to check an eventual masticatory muscles fibrosis. The MRI showed internal derangement of TMJ, bilateral mandibular condylar process resorption, and disk deformations with heterogeneous appearance (Figure 3 and Figure 4). The bilateral mandibular condylar process contains subchondral geodes and thinned cortical on sagittal and coronal views of MRI and hypomobility in open mouth. There was no fibrosis in masticatory muscles. The diagnosis of temporomandibular disorder (TMD) with arthralgia and myalgia related to SSc was made.

The patient was treated by an association of conservative techniques including rest, reassurance and jaw-opening exercises for painful joint. A myorelaxant was prescribed for 10 days to managing myofascial pain and aphysical therapy was indicated for 6 months including massage treatment on the bilateral masseter and temporal muscles, exercises, ultrasound, for improve jaw movements, managing musculoskeletal and myofacial pain. The patient was regularly reviewed monthly to evaluate pain and jaw movements. A great improvement in TMD symptoms was reported.

After 2 months of physical therapy, the mouth opening increased from 23 to 33 mm and a reduction in muscle pain was noted. The preauricular and muscle pain was gradually disappeared. The muscle flexibility has been partially improved by physiotherapy. However, a certain rigidity in the area of masseter muscles is still, due to its SSc and skin fibrosis. A relaxant nocturnal occlusal appliance was indicated to stabilize the results and was very supported by the patient.

## Discussion

SSc include a variety of oral and maxillofacial manifestations. In this case, we report an internal derangement of TMJ, disk deformations revealed in a patient with SSc. We also reported bilateral resorption of the mandibular condylar process which is uncommon but it has been reported previously in patients with SSc [4, 6–9]. In the study of Haers and Sailer [6], the mandibular areas bone resorption affected by SSc are mandibular angle that was most involved, followed by the condyle, the coronoid process and finally the posterior border of the ascending ramus. However, in a cohort of 30 consecutive patients, 30% had TMJ pain, but the radiological aspect of these TMJ was normal in 90% of cases and revealed a condylar erosion in only 10% [3]. Thus, the TMJ pain are not systematically correlated with a condylar resorption. The mechanism by which TMJ involvement is seen in patients with SSc is unclear. We suggested that inflammation or autoimmunity may cause the destruction of the capsular or disk attachment, resulting in internal derangement and subsequent degenerative joint diseases which result in articular surfaces changes in the condylar process and limited mouth opening. The possibility of primary articular surface resorption due to SSc is not excluded and it can result to internal derangement of TMJ which is a consequence of condylar resorption. It was suggested in the literature that the pathogenesis of primary TMJ bone lesions is due to both pressure ischemia originates from the rigidity and hardening of the overlying skin causing pressure and lack of mobility and vascular ischemia of small arterial branches of the internal maxillary artery caused by SSc [10].

However, there is no clear correlation between the incidence of the TMJ involvement and the severity, progression, and duration of SSc. In the present case, the TMJ signs was depicted 3 years after the initial diagnosis of SSc, but the symptoms appeared one year after the diagnosis of SSc.

## Conclusion

This case support the notion that TMJ manifestations of SSc must be considered by clinicians because their major functional impact and discomfort (limited jaw movements, pain,…). If the TMJ complications are diagnosed at late-stage, they will be very difficult to treat. Thus, it is recommended to regularly examine TMJ status with radiographic exploration in any patient with SSc to treat at time and avoid any complications.
